# Association of parental social support with energy balance-related behaviors in low-income and ethnically diverse children: a cross-sectional study

**DOI:** 10.1186/s12889-016-3829-8

**Published:** 2016-11-22

**Authors:** Natalia I. Heredia, Nalini Ranjit, Judith L. Warren, Alexandra E. Evans

**Affiliations:** 1 Center for Health Promotion and Prevention Research, The University of Texas Health Science Center at Houston (UTHealth) School of Public Health, 7000 Fannin St, Suite 2576E, Houston, TX 77030 USA; 2 Michael & Susan Dell Center for Healthy Living, The University of Texas Health Science Center (UTHealth) School of Public Health, Austin Regional Campus, Austin, TX USA; 3 Family & Community Health, Texas A&M AgriLife Extension Service, College Station, TX USA

**Keywords:** Social support, Physical activity, Nutrition, Minority populations, Child health

## Abstract

**Background:**

Parents play an important role in providing their children with social support for healthy eating and physical activity. However, different types of social support (e.g., instrumental, emotional, modeling, rules) might have different results on children’s actual behavior. The purpose of this study was to assess the association of the different types of social support with children’s physical activity and eating behaviors, as well as to examine whether these associations differ across racial/ethnic groups.

**Methods:**

We surveyed 1169 low-income, ethnically diverse third graders and their caregivers to assess how children’s physical activity and eating behaviors (fruit and vegetable and sugar-sweetened beverage intake) were associated with instrumental social support, emotional social support, modeling, rules and availability of certain foods in the home. We used sequential linear regression to test the association of parental social support with a child’s physical activity and eating behaviors, adjusting for covariates, and then stratified to assess the differences in this association between racial/ethnic groups.

**Results:**

Parental social support and covariates explained 9–13% of the variance in children’s energy balance-related behaviors. Family food culture was significantly associated with fruit and vegetable and sugar-sweetened beverage intake, with availability of sugar-sweetened beverages in the home also associated with sugar-sweetened beverage intake. Instrumental and emotional support for physical activity were significantly associated with the child’s physical activity. Results indicate that the association of various types of social support with children’s physical activity and eating behaviors differ across racial/ethnic groups.

**Conclusions:**

These results provide considerations for future interventions that aim to enhance parental support to improve children’s energy balance-related behaviors.

## Background

Childhood obesity continues to be a significant problem in the United States. Approximately 34% of children ages 6–11 are overweight or obese [1]. Low-income and minority children are disproportionately affected [2]; about 46% of Hispanics and 38% of non-Hispanic Blacks ages 6–11 years old are overweight or obese, as compared to 29% of non-Hispanic Whites [1]. Weight gain occurs when there is an imbalance between energy intake and energy expenditure. Lack of physical activity (PA) as well as overconsumption of energy-dense foods, such as sugar-sweetened beverages (SSB), can affect this balance and subsequent changes in body mass index (BMI) or adiposity [3–5]. These eating and PA behaviors are developed at a young age and typically track into adulthood, highlighting the need to address them earlier in the life span [6–9].

Parents’ influence on children’s PA and eating behaviors is exercised largely through the social support that they provide [10–15]. A variety of parental social support behaviors for children’s eating and PA have been identified, including instrumental and emotional support, modeling, having rules, and certain foods being available or unavailable at home [16–19]. Instrumental social support refers to tangible behaviors, and is illustrated, for example, by parents helping their child select and prepare healthy snacks or helping them do physical activity [18, 20–22]. Emotional support is intangible and is evident when parents provide encouragement for eating healthy foods or engaging in PA [23, 24], and by demonstrating these behaviors themselves, parents model proper eating or exercise to their children [17, 25, 26]. Setting rules about healthy eating, for example, what or how much of a specific food the child may have, is another form of parental support that can influence behavior [27]. Lastly, ensuring that fruits and vegetables (FV) are readily available in the home and that SSB are not has been shown to be a significant predictor of healthy eating [17, 28–31]. Each type of social support serves a different role and the impact on behavior can vary across the different types [23, 32, 33]. A better understanding of how the different types of social support contribute to children’s behaviors can help inform parenting practices and interventions targeting parenting practices [18, 23].

Although there is a wealth of research demonstrating associations between parental social support and children’s energy-balance and related behaviors, little is known about these associations among low socioeconomic status (SES) and minority children [12, 34]. The identified link between parental social support and children’s energy balance-related behaviors may be different in low SES communities, given the important influence of the built and the food environments and their difference between high and low SES groups [35–38]. Although some studies have demonstrated that the relationship holds in low SES and minority groups [39–42], few researchers have investigated the relative importance of the various types of parental social support in these communities or have explicitly examined ethnic/racial differences [43, 44]. For example, Donnelly and Springer, found that social support was significantly associated with vegetable intake in Hispanic children; this association was not found in White or African-American children [42]. More research is needed on how the various types of parental social support are associated with PA and eating behaviors among low SES and minority children.

The purpose of this study was to assess the association of various types of parental social support with a child’s PA and healthy eating in a sample of low SES, ethnically diverse third-grade students. Additionally, we determined how these associations varied across racial/ethnic categories. For this study, healthy eating was operationalized as more consumption of FV and less consumption of SSB. PA was operationalized as the number of times in the previous week children participated in sports, dance or played outdoor games during which they were very active.

## Methods

This study was approved by the University of Texas Health Science Center (HSC-SPH-10-0733) and the Texas A&M University Committees for the Protection of Human Subjects (2011–0012). The study was also approved by participating school districts’ Review Committees. Parents provided their written consent to participate, as well as written consent to let their child participate in the study. Students provided written assent at the time of data collection as well.

### Study design

This research examines the baseline data of the Texas Go! Eat! Grow! (TGEG) study of third-grade students and their parents in Texas. Additional details on the project and the protocol have been published elsewhere [45, 46]. Briefly, the goal of the 5-year TGEG study was to assess the independent and combined impact of gardening, nutrition and PA interventions on the prevalence of healthy eating, PA, and obesity status among low-income, third-grade students.

Researchers recruited 28 schools in 5 geographically distinct areas in Central Texas that met the following inclusion criteria: 1) classified as a Title I school, 2) located within the study’s geographical area, 3) were currently implementing the Coordinated Approach to Child Health program as a coordinated school wellness program [47, 48], 4) commitment at the district, principal, and teacher levels to participate, and 5) were willing to allow research staff to come into the school to recruit and collect data from third- and fourth-grade students. Third-grade students at these schools were recruited at the start of the fall 2012 and 2013 school years (the intervention was implemented using a split cohort). Eligible students were enrolled as third-grade students in the participating school at the time of baseline data collection. Students were excluded if they had a special diet or if English or Spanish was not their primary language. Parents or primary caretakers of third-grade students were included as long as they were able to read English or Spanish. Researchers administered baseline questionnaires to the child and the parent/caregiver. Consenting parents completed questionnaires at home, while students completed their questionnaires in the classroom during school hours and were provided a small incentive, such as a lunch bag or water bottle. The baseline questionnaire was completed by 1326 third graders and 1206 parents. A total of 1169 parent-child dyads completed the questionnaire at baseline in fall 2012 and 2013.

### Measures

The study measures are described below. Table 1 provides additional details on Cronbach’s α or Pearson’s *r* for the scales, response options, ranges, means and standard deviations for the social support variables. For all social support variables with more than one item, we calculated the scale score by multiplying the mean for the items in that variable by the number of items in that variable. All scales with 2 or more items demonstrated acceptable internal consistency [49, 50].

**Table 1:** Main independent variables

Source	Variable	# Items	Response options	Cronbach’s α	Pearson’s *r*	Potential Range	Actual Range	Mean (SD)
	Social Support for Healthy Eating							
Child	Family Food Culture	4	0 (Never or almost never) to 2 (almost always or always)	.64	NA	0–8	0–8	5.36 (1.80)
Parent	Instrumental support for healthy eating	7	0 (No) to 1 (Yes)	.76	NA	0–7	0–7	4.38 (2.04)
Parent	Home availability and accessibility of FV	6	0 (Never) to 3 (All of the time)	.72	NA	0–18	1–18	10.75 (3.57)
Parent	Home availability of SSB	1	0 (Never) to 3 (All of the time)	NA	NA	0–3	0–3	1.59 (.87)
Parent	Emotional support for healthy eating	6	0 (Strongly disagree) to 4 (Strongly agree)	.71	NA	0–24	0–24	17.66 (3.82)
Parent	Rules for healthy eating	3	0 (Strongly disagree) to 4 (Strongly agree)	.77	NA	0–12	0–12	8.96 (2.46)
Parent	Modeling vegetable intake	1	0 (Never) to 4 (About once a day)	NA	NA	0–4	0–4	3.48 (.84)
Parent	Modeling SSB	1	0 (Never) to 4 (About once a day)	NA	NA	0–4	0–4	2.79 (1.18)
	Social Support for Physical Activity							
Parent	Instrumental support for PA	2	0 (Never) to 7 (7 days)	NA	.46	0–14	0–14	3.96 (3.38)
Parent	Emotional support for PA	4	0 (Strongly disagree) to 4 (Strongly agree)	.74	NA	0–16	0–16	12.73 (2.52)
Parent	Modeling PA	1	0 (Never) to 4 (About once a day)	NA	NA	0–4	0–4	3.55 (.82)

#### Social support for healthy eating

We assessed family food culture using a four-question scale specifically developed for this study that asked children the following: how often they eat breakfast, eat evening meals, go out to eat, and help prepare food with their families. We measured instrumental support for healthy eating using an adapted scale of seven questions from a previously validated measure that asked if parents did several different diet-related activities with their child the previous week, including buying vegetables that their child liked or helping their child make a snack that included vegetables [51].

Home availability and accessibility of FV was assessed by asking parents six questions about whether 100% fruit juice, vegetable juice, fresh vegetables, frozen or dried vegetables, salad and cut-up fresh vegetables were available in the home during the previous week [29]. We assessed home availability of SSB with a single question asking parents how often soft-drinks or SSB were available in the home in the previous week.

We asked parents six questions to measure emotional support for healthy eating with example statements such as, “I show approval when my child eats what I want her/him to eat” and “I encourage my child to try new foods.” Rules for healthy eating were assessed with three questions about parents’ control of intake of sweets, high fat foods, and what the child eats away from home. We measured modeling of vegetable and SSB intake by asking parents how often their child saw them eating vegetables and drinking SSB.

#### Social support for physical activity

We assessed parental modeling of PA with one question that elicited how often the child sees the parent being active. We measured instrumental support for PA with two questions gauging how many days per week parents went for a walk or did other PA with their child and emotional support with four questions that determined how much they encourage, watch, and show approval for PA.

#### Fruit and vegetable intake

Children self-reported their FV intake using previously validated measures [52–54]. We asked them if they drank 100% fruit juice and if they ate fruit, orange vegetables, salads, or other vegetables during the previous day. We used a Likert-like scale for these questions with 0 indicating “No, I didn’t eat/drink any of these yesterday” and 3 indicating “Yes, I ate/drank × 3 or more times yesterday.” We aggregated the responses to the five questions to determine the child’s total FV intake the previous day.

#### Sugar-sweetened beverage intake

Children self-reported their SSB intake in two questions, 1) if they had consumed any punch, Kool-Aid, sports drinks, or other fruit flavored drinks the previous day and 2) if they drank any regular sodas or soft drinks the previous day. Answers were on a Likert-like scale with 0 indicating “No, I didn’t drink any of these yesterday” and 3 indicating “Yes, I drank × 3 or more times yesterday.” We aggregated the responses to get the child’s total SSB intake for the previous day.

#### Physical activity

Parents reported how many times in the previous week their child engaged in sports, dance or outdoor play, outside of school. Response options ranged from 0 indicating “None” to 4 indicating “6 or more times.”

#### Demographics

Children self-reported their age and gender; parents self-reported their gender, relationship to the child, age, race, ethnicity, employment status, highest level of education, and marital status. Food insecurity was measured on a scale from “almost always” to “almost never or never” by asking parents “How often do you run out of food before the end of the month because you can’t afford to buy more?” [55]. Parents were asked what language was spoken at home with answer choices of English, Spanish, or Other. They were also asked if the family received federal benefits, such as the Supplemental Nutrition Assistance Program (SNAP) and The Special Supplemental Nutrition Assistance Program for Women, Infants, and Children (WIC), and whether their child received a free or reduced-cost school lunch.

#### Anthropometric measures

Height and weight were collected during school site visits by two project staff members who were trained by the program director and certified for essential skills [45]. Height was measured using the Perspective Enterprise Model PE-AIM-10 stadiometers and weight using the Tanita scale model BWB-800S. BMI was calculated from height and weight data, and the students were placed into BMI categories using growth charts from the Centers for Disease Control and Prevention [56].

### Data analysis

Preliminary descriptive analyses were conducted by examining frequency distributions of key demographic variables in the sample. The levels of the different types of parental social support for eating and PA behavior were compared across the demographic categories (gender, BMI, race/ethnicity) of children using independent samples t-test or one-way ANOVA, as appropriate. We then used sequential linear regression with listwise deletion to assess the relationship between social support variables and FV intake, SSB intake, and PA, while controlling for important covariates, including race/ethnicity variables, gender, BMI z-score, food security, receiving free or reduced-cost lunch, and parental education [57–59]. For all three energy balance outcomes, we entered child’s gender, BMI, race/ethnicity, receiving free or reduced-cost lunch, and parental education into step 1, food insecurity into step 2, and the social support variables into step 3. The threshold for significance was set at *p* < .05.

## Results

### Sample characteristics

There were 1169 parent-child dyads included in this study (Table 2). Children were third-grade students in Texas, between the ages of 7 and 11. Students were 42% female, 33% Hispanic, and 74% received free or reduced-cost lunch. Of the parents and caregivers, 83% were female. Almost 92% of caregivers indicated they were a parent, while 5% indicated they were a grandparent or other caregiver, and 3% were missing (not shown in table). About 51% of parents had a high school diploma, GED, or less education. In our sample, 42% of families indicated that they received SNAP, 12% received WIC, and 41% said that the family sometimes or almost always experienced food insecurity.

**Table 2:** Participant demographics, full sample

	Number	Percent
Child demographics	1169	100
*Gender*		
Male	495	42.3
Female	492	42.1
Missing	182	15.6
*Age*		
7 years old	6	.5
8 years old	672	57.5
9 years old	269	23.0
10 years old	24	2.0
11 years old	3	.3
Missing	195	16.7
*Race/Ethnicity*		
White	209	17.9
Black	179	15.3
Hispanic	385	32.9
Other	204	17.5
Missing	192	16.4
*Weight status*		
Underweight	27	2.3
Normal Weight	466	39.9
Overweight	171	14.6
Obese	270	23.1
Missing	235	20.1
Parent demographics	1169	100
*Gender*		
Male	132	11.3
Female	970	83.0
Missing	67	5.7
*Age*		
Less than 30	219	18.8
30 to 34	336	28.7
35 to 39	211	18.0
40 and above	246	21.1
Missing	157	13.4
*Employment status*		
Full-time	557	47.7
Part-time	157	13.4
No work outside the home	372	31.8
Retried	13	1.1
Missing	70	6.0
*Education*		
Less than 12 years	231	19.8
High school or GED	360	30.8
Trade/Tech college	100	8.5
Some college	206	17.6
College or advanced degree	173	14.8
Missing	99	8.5
*Marital status*		
Married	634	54.2
Separated or Divorced	178	15.2
Single, never married	264	22.6
Widowed	25	2.2
Missing	68	5.8
Family demographics	1169	100
*Language spoken at home*		
English	786	67.2
Spanish	295	25.2
Other	16	1.4
Missing	72	6.2
*Food insecurity*		
Almost never or never	625	53.5
Sometimes	331	28.3
Almost always	152	13.0
Missing	61	5.2
*Child receives free or reduced lunch*		
Yes	861	73.7
No	240	20.5
Missing	68	5.8
*SNAP recipients*		
Yes	494	42.3
No	591	50.5
Missing	84	7.2
*WIC recipients*		
Yes	140	12.0
No	945	80.8
Missing	84	7.2

### Level of parental support by sex, race/ethnicity, and weight status

There was a significant difference between boys and girls for family food culture and instrumental support for healthy eating, with girls having a higher mean for both (Table 3). There were also significant differences between racial/ethnic groups for home availability and accessibility of FV, emotional support for healthy eating, rules for eating, modeling of vegetable intake and modeling SSB intake (Table 3). Black children had a higher mean for home availability and accessibility of FV and rules for eating compared to the other three groups. White children had the highest mean for emotional support for healthy eating and modeling of vegetable intake, while Hispanic children had the highest mean for modeling SSB intake. Lastly, there were also significant differences by child's weight status for emotional support for healthy eating and modeling of vegetable intake (Table 3). Interestingly, overweight children had the highest mean for emotional support for healthy eating and normal weight children had the highest mean for modeling of vegetable intake.

**Table 3:** Level of parental social support for eating and physical activity behavior by group

	Family Food Culture	Instrumental support for healthy eating	Home availability/accessibility of FV	Home availability of SSB	Emotional support for healthy eating	Rules for eating	Modeling vegetable intake	Modeling SSB intake	Instrumental support for PA	Emotional support for PA	Modeling of PA
	*N* = 977	*N* = 1148	*N* = 1164	*N* = 1151	*N* = 1146	*N* = 1145	*N* = 1143	*N* = 1139	*N* = 1139	*N* = 1127	*N* = 1144
Boys, Mean (SD)	5.23 (1.86)	4.13 (2.03)	10.56 (3.63)	1.58 (.86)	17.46 (3.88)	8.91 (2.42)	3.47 (.82)	2.80 (1.17)	3.86 (3.26)	12.84 (2.50)	3.54 (.81)
Girls, Mean (SD)	5.48 (1.73)	4.60 (1.99)	10.81 (3.56)	1.59 (.88)	17.68 (3.77)	8.95 (2.48)	3.49 (.80)	2.75 (1.23)	4.02 (3.32)	12.63 (2.52)	3.55 (.81)
*t* ^a^	−2.18*	. − 3.59***	−1.09	−.24	−.91	−.24	−.28	.71	−.743	1.27	−.17
White, Mean (SD)	5.12 (1.79)	4.30 (1.94)	10.90 (3.26)	1.64 (.90)	18.24 (3.13)	8.68 (2.33)	3.65 (.69)	2.73 (1.28)	3.53 (3.07)	12.98 (2.35)	3.60 (.66)
Black, Mean (SD)	5.53 (2.00)	4.44 (1.89)	11.57 (3.70)	1.62 (.86)	17.96 (3.70)	9.30 (2.49)	3.61 (.66)	2.66 (1.14)	4.14 (3.58)	13.02 (2.49)	3.51 (.87)
Hispanic, Mean (SD)	5.46 (1.74)	4.42 (2.12)	10.29 (3.69)	1.54 (.82)	16.99 (4.15)	8.79 (2.43)	3.36 (.86)	2.92 (1.12)	4.14 (3.24)	12.51 (2.51)	3.55 (.86)
Other, Mean (SD)	5.26 (1.68)	4.30 (2.04)	10.43 (3.47)	1.58 (.95)	17.65 (3.79)	9.12 (2.55)	3.42 (.91)	2.62 (1.28)	3.92 (3.33)	12.69 (2.65)	3.50 (.81)
*F* ^b^	2.40	.31	5.84**	.77	5.72**	2.88*	7.78***	3.77*	1.71	2.46	.73
Under weight, Mean (SD)	5.30 (1.66)	4.33 (2.00)	11.31 (3.81)	1.59 (1.05)	17.98 (3.08)	9.46 (2.48)	3.42 (.95)	2.67 (1.21)	4.15 (3.76)	12.87 (2.45)	3.44 (.85)
Normal weight, Mean (SD)	5.49 (1.82)	4.51 (2.03)	10.77 (3.54)	1.55 (.88)	17.79 (3.76)	9.06 (2.43)	3.56 (.74)	2.81 (1.18)	4.13 (3.37)	12.73 (2.52)	3.59 (.78)
Overweight, Mean (SD)	5.32 (1.83)	4.40 (1.94)	10.89 (3.54)	1.69 (.87)	18.02 (3.88)	8.82 (2.64)	3.47 (.80)	2.78 (1.18)	3.77 (3.46)	13.13 (2.47)	3.56 (.81)
Obese, Mean (SD)	5.36 (1.78)	4.08 (2.07)	10.34 (3.75)	1.55 (.81)	16.82 (3.85)	8.82 (2.36)	3.38 (.92)	2.73 (1.24)	3.79 (3.01)	12.52 (2.52)	3.47 (.88)
*F* ^b^	.13	2.48	1.38	1.23	4.77**	1.08	2.82*	.29	.88	1.95	1.36

Note: *p < 0.05, **p < 0.01, ***p < 0.001

^a^Independent-samples *t*-test, ^b^One-way ANOVA

### Associations between parental social support and healthy eating

After adjusting for covariates the sequential regression showed that of the social support variables, only family food culture was significantly associated with FV intake (Table 4). BMI z-score and receiving free or reduced-cost lunch were also significantly associated with FV intake.

**Table 4:** Sequential regression analysis for association of social support with FV intake

	*B*	*SE B*	*β*	*R* ^*2*^	*R* ^*2*^ *change*
Step 1				.055***	.055***
Gender	−.413	.265	−.054		
BMI z-score	.306	.109	.097**		
Receive free or reduced lunch	.949	.38	.104*		
Parent’s education	−.184	.101	−.072		
Black	.779	.435	.078		
Hispanic	.167	.385	.021		
Other	.723	.411	.077		
Step 2				.055***	.000
Food insecurity	.158	.287	.021		
Step 3				.128***	.073***
Family food culture	.5597	.074	.259***		
Instrumental support for healthy eating	.037	.073	.020		
Home availability/accessibility of FV	.057	.044	.053		
Home availability of SSB	−.165	.171	−.038		
Emotional support for healthy eating	−.036	.039	−.036		
Rules for eating	−.076	.062	−.050		
Modeling of vegetable intake	−.068	.176	−.014		
Modeling of SSB intake	−.040	.124	−.012		

Note: *B* = Unstandardized beta coefficient; *SE B* = Standard error for *B*; *β* = Standardized beta coefficient; *R* ^*2*^ = adjusted R-square; *p < 0.05; **p < 0.01; ***p < 0.001

For SSB intake, both family food culture and home availability of those beverages were significantly associated with their intake, as were gender and free or reduced-cost lunch (Table 5). The social support variables and sociodemographic covariates explained about 13% of the variance in FV intake and 9% of the variance in SSB intake.

**Table 5:** Sequential regression analysis for association of social support with SSB intake

	*B*	*SE B*	*β*	*R* ^*2*^	*R* ^*2*^ *change*
Step 1				.053***	.053***
Male	−.457	.124	−.130***		
BMI z-score	−.003	.051	−.002		
Receive free or reduced lunch	.367	.180	.088*		
Parent’s education	−.074	.047	−.063		
Black	.405	.205	.087*		
Hispanic	.091	.180	.025		
Other	.050	.191	.012		
Step 2				.056***	.003
Food insecurity	.171	.135	.048		
Step 3				.086***	.030**
Family food culture	.098	.035	.098**		
Instrumental support for healthy eating	.052	.034	.061		
Home availability/accessibility of FV	−.002	.021	−.004		
Home availability of SSB	.187	.080	.093**		
Emotional support for healthy eating	.012	.018	.025		
Rules for eating	−.035	.029	−.049		
Modeling of vegetable intake	−.114	.082	−.053		
Modeling of SSB intake	.028	.058	.019		

Note: *B* = Unstandardized beta coefficient; *SE B* = Standard error for *B*; *β* = Standardized beta coefficient; *R* ^*2*^ = adjusted R-square; *p < .05; **p < .01; ***p < .001

### Associations between parental social support and physical activity

After adjusting for covariates, both instrumental and emotional support for PA were significantly associated with the child’s PA (Table 6). The social support variables and the sociodemographic covariates explained about 13% of the variance in child’s PA the previous week.

**Table 6:** Sequential regression analysis for association of social support with physical activity

	*B*	*SE B*	*β*	*R* ^*2*^	*R* ^*2*^ *change*
Step 1				.006	.006
Gender	−.112	.077	−.049		
BMI z-score	.009	.032	.009		
Receive free or reduced lunch	.002	.111	.001		
Parent’s education	−.003	.030	−.004		
Black	−.033	.126	−.011		
Hispanic	−.143	.111	−.061		
Other	−.010	.118	−.003		
Step 2				.006	.000
Food insecurity	.080	.084	.035		
Step 3				.130***	.124***
Instrumental support for PA	.093	.013	.264***		
Emotional support for PA	.062	.016	.137***		
Modeling of PA	.086	.050	.062		

Note: *B* = Unstandardized beta coefficient; *SE B* = Standard error for *B*; *β* = Standardized beta coefficient; *R* ^*2*^ = adjusted R-square; ***p < 0.001

### Stratification by race and ethnicity

Stratifying by race/ethnicity demonstrated some differences in the relationship between social support and the energy balance-related behaviors between racial/ethnic groups (Table 7). The association between all social support variables and FV intake was not significant in White children, but the models were significant for all other racial/ethnic groups. Emotional support was significantly associated with FV intake in Black children, but in no other group. Within significant models, family food culture was significantly associated with SSB intake in White children, home availability of those beverages was significantly associated with their intake only in Hispanics and Others, and instrumental support for healthy eating was significant only in Hispanic children. The association between social support and SSB intake, as well as social support and PA, were not significant in Black children. Instrumental support was significantly associated with a child’s PA for Hispanic and Other, but not for White children. Emotional support for PA was significantly related to child’s PA for both Hispanic and White children, and parental modeling of PA was significantly associated with PA behaviors only for White children.

**Table 7:** Association of social support with eating and physical activity, stratified by racial/ethnic group

	White			Black			Hispanic			Other		
	*R* ^*2*^	*B*	*SE*	*R* ^*2*^	*B*	*SE*	*R* ^*2*^	*B*	*SE*	*R* ^*2*^	*B*	*SE*
*FV Intake* ^a^	*.112*			.202**			.126***			.189**		
Family food culture		.400*	.154		.691***	.181		.622***	.125		.421*	.176
Instrumental support for healthy eating		−.126	.150		.125	.209		.058	.115		.227	.166
Home availability and accessibility of FV		−.007	.096		−.106	.120		.050	.069		**.**154	.101
Home availability of SSB		−.303	.325		.277	.479		−.232	.286		−.038	.402
Emotional support for healthy eating		−.027	.097		−.212*	.107		−.027	.058		−.012	.091
Rules for eating		−.016	.134		.042	.151		−.064	.101		−.203	.148
Modeling of vegetable intake		−.116	.424		1.150	.637		−.045	.273		−.462	.336
Modeling of SSB intake		.102	.241		−.528	.352		.123	.210		−.037	.283
*SSB Intake* ^a^	.143*			*.116*			.103**			.140*		
Family food culture		.169*	.073		.188*	.086		.087	.059		−.010	.081
Instrumental support for healthy eating		−.039	.072		.054	.095		.113*	.055		.134	.079
Home availability and accessibility of FV		.033	.045		−.010	.055		−.021	.033		−.025	.046
Home availability of SSB		.115	.153		−.250	.219		.311*	.137		.374*	.177
Emotional support for healthy eating		.048	.046		.016	.049		.006	.027		−.026	.040
Rules for eating		−.044	.062		−.036	.073		−.064	.048		−.002	.067
Modeling of vegetable intake		−.015	.200		.247	.296		−.132	.127		−.272	.156
Modeling of SSB intake		−.066	.113		.071	.169		.023	.099		.075	.124
*PA last week* ^a^	.137**			*.085*			.173***			.188***		
Instrumental support PA		.008	.031		.085**	.030		.115***	.020		.126***	.029
Emotional support PA		.090*	.038		.050	.044		.068**	.025		.045	.035
Modeling of PA		.436**	.142		−.014	.122		.038	.071		.046	.115

Note: bold numbers are only used for models that are significant

*B* = Unstandardized beta coefficient; *SE* = Standard error for *B*; *R* ^2^ = adjusted R-square; *p < .05; **p < .01; ***p < .001

^a^Covariates: gender, BMI z-score, free or reduced lunch, parent’s education, food insecurity; not pictured

## Discussion

The sample of the TGEG study with third graders in Texas was largely composed of minority (Hispanic and non-Hispanic Black) children. Of our sample, 47.2% were overweight or obese, which is 13% higher than the U.S. prevalence for children 6–11 years of age [1]. The sample had high values for modeling of vegetable intake, modeling PA, and emotional support for PA while most other variables had averages that fell in the third quartile of the range (Table 1). It is possible that parents in this sample felt capable and were already providing emotional social support for physical activity and were themselves participating in PA and consuming more vegetables, making modeling for these behaviors easier. However, instrumental support for PA was low as compared to the other scales, likely because it was the only scale measuring the number of days parents actually provided a specific type of support for their child. This study showed that while there was minimal difference in the various types of social support that girls and boys received, there were some meaningful differences between racial groups for certain types of social support. Of the racial and ethnic groups, Hispanic children reported substantially lower levels of home availability and accessibility of FV and emotional support for eating those foods, as compared to other racial/ethnic groups. Researchers previously identified lower levels of social support in this group [42, 60]. Our findings further highlight the importance of explicitly addressing these disparities in social support when developing interventions targeting Hispanic parents, potentially with additional skills training or increased intervention doses. There were also differences in parental social support based on the child’s weight status; for example, overweight children received more emotional support for healthy eating. However, in contrast to previous studies that suggest that overweight and obese children receive less parental support for PA [61, 62], we found no differences in social support by child's weight status.

We found some other associations between parental social support and energy balance-related behaviors in children to be consistent with the literature, such as the association of instrumental [63–65] and emotional support for PA with PA behavior in children [23, 66, 67]. Home availability of SSB was significantly associated with SSB intake, as seen in earlier research [30, 68, 69]. However, we noted differences from previous studies. It was unexpected that home availability and accessibility of FV was not associated with FV intake, as the association has been reported previously [29, 70–72]. Similarly, it was surprising to find no association of instrumental or emotional support for healthy eating, modeling vegetable intake, and modeling PA with the outcomes, as these types of support have been found to be associated with children’s energy balance-related behaviors in other populations [11, 71–75]. Family food culture was associated with FV intake, consistent with the literature that shows that increased family meals, the main component of family food culture, is associated with increased FV intake in children [76–79]. This was the only significant variable in the FV intake model and also the only child-reported social support; other studies have also found that various types of parental support reported by children were more associated with children’s FV intake than the parent’s perceptions of that same support [80–82].

We also found associations between some of the social support variables and behavioral outcomes that were in unexpected directions. In the case of the positive association of family food culture with SSB intake, it could be that the current family food culture is generally unhealthy [83, 84]. The unexpected associations could be a result of the influence of other variables, such as family cohesion [85, 86], or could demonstrate the child’s rebellion against parents if the social support is perceived as a demand for behavior change [10]. It is possible that these unexpected findings may also indicate that parents are not the most important source for social support. In fact, many researchers report that peer social support might be more influential than parental support for many of these energy balance-related behaviors [23, 32, 33, 87]. However, more research is needed in this area because parental social support has been identified as an important factor for energy balance-related behaviors in children [17, 18].

Several social support variables were significantly associated with energy balance-related behaviors in certain groups but not in others, demonstrating potential differences in the relative impact of parental social support on children’s subsequent behaviors. Given the differences, there could be implications for intervention development. For example, emphasizing emotional support for healthy eating in Black families may lead to greater changes in FV intake than a focus on other types of support. Given the importance of home availability of SSB on the SSB intake of Hispanic children, this should be one place of emphasis for interventions targeting Hispanic parents. However, an intervention with White parents with a similar target of reducing SSB intake may aim to alter the family food culture instead. For PA, interventionists may consider focusing on building instrumental and emotional support skills for PA among Hispanic parents. The insignificant models among Black children for both PA and SSB intake might indicate that other external factors in their environment [35–38] reduce the relative importance of parental social support for those energy-balance related behaviors and thus interventionists may consider looking elsewhere for the first point of intervention. As receiving free or reduced lunch at school was associated with FV intake in the overall model and the stratified model for Black children (data not shown), ensuring children have access to these programs might be more critical.

### Limitations

Given the cross-sectional nature of our data, it is unclear how parental social support can causally impact children’s eating and PA behaviors, as a determination about temporality could not be made and there remains the possibility of reverse causality. Our modeling variables and home availability of SSB were one item scales, limiting how well we could capture these constructs and the conclusions that can be drawn. We were limited in the information that could be accurately collected from the children, thus most variables relied on the parental report of social support or children’s behavior. Lastly, we have not previously done extensive reliability and validity testing for some of the measures developed for this study, which may impact results. However, the Cronbach’s alphas for the items in the scales were acceptable, indicating that the scales had good internal consistency. Despite these limitations, the findings offer greater insights into the relative association of different types of parental social support with energy-balance behaviors among low-income and diverse children.

## Conclusions

Few studies have looked at parental social support and energy balance-related behaviors across racial and ethnic groups or made comparisons [39–42, 60]. This study is one of the few to compare the association of various types of parental social support and energy balance-related behaviors in children across racial and ethnic groups and provides evidence that the associations may differ between racial and ethnic groups. Future studies should attempt to assess the longitudinal relationship of parental social support with children’s energy balance-related behaviors as well as the individual importance of each type of social support. Researchers developing interventions that impact parents to ultimately improve energy balance in children should take into account the types of social support most associated with the behavior of interest in their target population.

## Abbreviations

BMI: Body mass index
F&V: Fruits and vegetables
PA: Physical activity
SES: Socioeconomic status
SNAP: Supplemental Nutrition Assistance Program
SSB: Sugar - sweetened beverages
TGEG: Texas Grow! Eat! Go!
WIC: The Special Supplemental Nutrition Assistance Program for Women, Infants, and Children
